# Using financial incentives to support service engagement of adults experiencing homelessness and mental illness: A qualitative analysis of key stakeholder perspectives

**DOI:** 10.1111/hex.13442

**Published:** 2022-02-01

**Authors:** Nadine Reid, Rebecca Brown, Cheryl Pedersen, Nicole Kozloff, Alexandra Sosnowski, Vicky Stergiopoulos

**Affiliations:** ^1^ General and Health Systems Psychiatry Division Centre for Addiction and Mental Health Toronto Ontario Canada; ^2^ Health Services Research Program, Institute of Health Policy, Management and Evaluation University of Toronto Toronto Ontario Canada; ^3^ MAP Centre for Urban Health Solutions, St. Michael's Hospital Unity Health Toronto Toronto Ontario Canada; ^4^ Department of Psychiatry University of Toronto Toronto Ontario Canada; ^5^ Child, Youth and Emerging Adult Program Centre of Addiction and Mental Health Toronto Ontario Canada

**Keywords:** financial incentives, health services research, homelessness, mental illness, qualitative, service engagement

## Abstract

**Introduction:**

Homelessness and mental illness are associated with poor service engagement, health and health service use outcomes. Existing literature suggests that financial incentives may effectively support service engagement of this population, but studies investigating key stakeholder perspectives are lacking. This study aimed to elicit, using qualitative methods, nuanced service user and provider experiences by using financial incentives to support service engagement among adults experiencing homelessness and mental illness.

**Methods:**

This qualitative study is part of a larger mixed‐methods pragmatic trial of financial incentives (Coordinated Access to Care for the Homeless—Financial Incentives [CATCH‐FI]) within a community‐based brief case management programme (CATCH) in Toronto, Ontario. Twenty‐two CATCH‐FI participants were purposefully recruited to participate in in‐depth, semi‐structured interviews; five CATCH service providers participated in a focus group and seven key informants in individual interviews. Data collection occurred between April 2019 and December 2020. All interviews and the focus group were audio‐recorded and transcribed. Topic guides prompted participant perspectives on and experiences of using financial incentives to support engagement, health and well‐being. Grounded theory and inductive thematic analysis guided coding and interpretation of transcripts. Triangulation and member‐checking enhanced the analytical rigour and validity of findings.

**Results:**

CATCH service providers, key informants and subgroup of CATCH‐FI participants perceived financial incentives to directly facilitate service engagement. The majority of CATCH‐FI participants however highlighted that intrinsic motivation and service quality may be relatively more important facilitators of engagement. Most study participants across stakeholder groups perceived that financial incentives have direct positive influences on health and well‐being in enabling access to basic needs and simple pleasures.

**Conclusions:**

Our data suggest that for some adults experiencing homelessness and mental illness, financial incentives can directly support service engagement. In addition, financial incentives may positively impact health and well‐being by easing financial stress and enabling deeper attention to individual health needs. Further research on the effectiveness and acceptability of financial incentives is needed to improve understanding and uptake of a promising intervention to support health and health service use outcomes in an underserved population.

**Patient or Public Contribution:**

Study participants provided input into the study research questions, study design, interview guides and interpretation of findings.

## 1 INTRODUCTION

Adults experiencing homelessness and mental illness face significantly worse health and health outcomes compared to the general population, including increased prevalence and severity of chronic health conditions, comorbid alcohol and substance use disorders, neurocognitive impairment and premature mortality.^1^, ^2^, ^3^ Engaging this population in health services is challenging due to transiency in housing, complex health needs, financial barriers, limited availability of tailored and appropriate services and stigma and discrimination.^4^, ^5^, ^6^, ^7^, ^8^ In addition, the literature on interventions to improve service engagement among adults experiencing homelessness and mental illness is limited. As low levels of engagement are associated with a range of poor outcomes, including greater illness severity, lower quality of life and higher rates of acute care use,^6^, ^9^, ^10^ implementing strategies to improve service engagement of this population remains a priority across healthcare and social service settings.

A review of existing literature suggests that person‐centred care and a strong therapeutic alliance within a recovery‐oriented framework are helpful in supporting service engagement in people experiencing mental illness.^6^ Studies also indicate that providing instrumental supports and services that attend to immediate needs, such as financial, housing and employment assistance, in addition to direct mental healthcare, are important for this population.^11^, ^12^

Financial incentives (FIs) in particular have been used successfully to influence health decisions in a variety of populations and healthcare settings.^13^, ^14^, ^15^, ^16^ It has been suggested that FIs may be particularly effective in facilitating service engagement of underserved populations in shorter‐term interventions.^17^, ^18^, ^19^ For people experiencing homelessness and/or mental illness, existing literature suggests that FIs have been successfully implemented to improve attendance in psychotherapy services ^20^, ^21^ and to increase rates of medication adherence,^22^ abstinence from substances^23^, ^24^ and smoking cessation.^25^, ^26^

In healthcare, behavioural economics suggests that one way in which FIs may effectively influence individuals' health decision‐making is by appealing to our general tendency, as humans, to focus on the present and on immediate gratification versus future‐oriented pay‐outs.^17^, ^27^, ^28^ Motivation theories, including self‐determination theory,^29^ also align with this notion and further suggest that incentive‐based interventions may be relatively more effective among extrinsically motivated individuals.^30^ While the literature has long‐identified intrinsic motivation as central to sustained health behaviours,^29^, ^31^, ^32^, ^33^ little is known regarding the extent to which and how the experience and impact of FIs might differ within and across populations and settings.

Despite growing evidence of effectiveness, there is limited research on the acceptability of FIs, or a nuanced understanding of stakeholder perspectives on the impact of FIs on health service engagement in underserved populations. Moreover, significant ethical concerns have been raised, particularly regarding perceived coercion and its potential impact on autonomous decision‐making, and the potential for unintended harms; service providers, researchers and planners have raised concerns that money might enable increased substance use, for example.^34^, ^35^, ^36^, ^37^, ^38^ Given debated appropriateness of FIs to promote service engagement, more evidence is needed to better understand key stakeholder perspectives related to impact, utility and ethicality, specifically including perceived impact on autonomy and unintended consequences, which together with evidence of effectiveness, can help inform acceptable implementation in practice.

This study aimed to elicit, using qualitative methods, nuanced service user and provider experiences with financial incentives to support service engagement among adults experiencing homelessness and mental illness posthospital discharge in Toronto, Canada.

## 2 MATERIALS AND METHODS

### 2.1 Intervention description

This study describes the qualitative component of a larger mixed‐methods pragmatic randomized controlled trial (RCT), Coordinated Access to Care for the Homeless—Financial Incentives (CATCH‐FI), described in‐depth elsewhere.^39^ This RCT aimed to evaluate the impact of FI on service engagement among adults experiencing homelessness and mental illness posthospital discharge in Toronto, Canada. The CATCH‐FI study enroled and randomly assigned participants of a brief case management programme, Coordinated Access to Care for the Homeless (CATCH), to either: (i) an intervention arm, in which participants received a $20 FI for each week they contacted their CATCH case manager or another CATCH service provider (up to $80 per month) over a 6 months follow‐up period or until they were discharged from the programme according to their care plan; or (ii) a control arm, in which participants received usual CATCH care without an FI to support engagement with the programme.

The CATCH programme itself has also been extensively described and evaluated.^40^, ^41^, ^42^, ^43^, ^44^, ^45^ In short, CATCH is a multidisciplinary brief case management intervention that bridges multiple organisations and sectors to provide comprehensive, short‐term support to individuals experiencing homelessness and mental illness and transitioning from hospital to community services. The programme, informed by the critical time intervention model,^46^ is associated with significant positive changes in physical and mental health, health service use and quality of life.^12^, ^40^, ^41^, ^43^, ^47^

### 2.2 Study participants and recruitment

CATCH‐FI participants in this qualitative study were recruited from the broader RCT sample (*N* = 176) and met both programme and RCT eligibility criteria. Programme eligibility included: (i) current homelessness or precarious housing^39^; (ii) service provider‐determined unmet health needs; (iii) service user‐determined unmet support needs; and (iv) aged 18 years or older. Exclusion criteria included aggression or illness severity requiring higher intensity supports. For CATCH clients to enrol in the RCT, additional eligibility included: (i) new programme referral; (ii) recently admitted or readmitted to hospital services; and (iii) completed programme intake.

Thirty‐four participants were recruited for this qualitative study, a sample size sufficient for achieving thematic saturation within each stakeholder group. Twenty‐two CATCH‐FI participants were purposefully selected based on the number of contacts with the CATCH team, sociodemographic representativeness, group assignment and study staff's assessment of participants' ability to provide in‐depth reflections on their experiences. Eligibility criteria and recruitment efforts were adapted throughout to improve the representativeness of the study sample; for example, efforts were made to include participants with low levels of engagement and minority groups. This recruitment approach has been used by the research team in previous studies among people experiencing homelessness and mental illness^48^, ^49^ and within this particular programme.^40^ In addition to CATCH‐FI participants, 12 CATCH service providers and key informants were purposefully recruited based on their role in the CATCH programme or the local healthcare system.

At baseline, most CATCH‐FI participants (*n* = 22) had at least 10 contacts with their CATCH service providers (*n* = 13; 59%). CATCH‐FI participants were predominantly male (*n* = 15; 68%), Caucasian (*n* = 15; 68%) and middle‐aged, with 75% aged 35–64 years. CATCH‐FI participants varied in level of education but the majority had completed high school (*n* = 16; 73%). Compared to the overall RCT sample, this qualitative sample was comparable in age but included proportionally more female (32% vs. 25%), Caucasian (68% vs. 56%) and high‐school educated (73% vs. 64%) participants.

CATCH service providers and key informants ranged in age, gender, experience and healthcare role. Focus group participants (*n* = 5) were CATCH programme staff in frontline (case management, nursing) and managerial roles. Key informants (*n* = 7) were healthcare providers in senior clinical and/or administrative leadership roles at leading local mental health and social service institutions that are partnered with the CATCH programme and serve the broader homeless population in community settings.

### 2.3 Data collection

In‐depth, semi‐structured qualitative interviews averaging 40 min in length (range: 15–111 min) were conducted with CATCH‐FI participants between April 2019 and December 2020. Key informant interviews averaging 26 min in length (range: 15–51 min) took place between August 2020 and October 2020, while a focus group with CATCH service providers was held in August 2020, lasting 94 min. All interviews including the focus group were audio‐recorded and transcribed verbatim. CATCH‐FI participants received an honorarium of $30 and public transportation fare for completing a qualitative interview and focus group participants received a $10 gift card.

The individual interviews and focus group specifically aimed to explore stakeholder perspectives and experiences related to the impact of FI on service engagement and health and well‐being. Topic guides included open questions and specific prompts related to overall perceived facilitators of and barriers to engagement and factors influencing healthcare decisions, as well as specific questions and prompts related to the perceived impact of FIs on service engagement, health and well‐being. More specifically, CATCH‐FI participants enroled in the intervention arm were asked whether FI impacted their decision to contact their case manager or their overall health and well‐being and how. Those enroled in the usual care arm were asked whether they believed FI would have, hypothetically, impacted their engagement, health and well‐being. Topic guides were iteratively developed and refined by the study team to capture rich and diverse perspectives.

The research team is led by a primary investigator (PI) with extensive experience conducting qualitative research among people experiencing homelessness and mental illness; and the study is sponsored by the MAP Centre for Urban Health Solutions at Unity Health Toronto, who similarly have a history of successfully engaging this population locally. Together, this team has successfully conducted several previous studies with the study population. Data collection was conducted by trained and experienced interviewers who were known to participants by virtue of having conducted prior quantitative interviews as part of the broader RCT. Rigorous interviewer training, ongoing transcript review by the study PI and study staff and investigator triangulation were also employed to strengthen the quality, rigour and trustworthiness of results. Lastly, a member‐checking process was conducted in which results were reviewed, refined and confirmed with CATCH service providers in August 2021.

### 2.4 IRB statement

This study was approved by the Research Ethics Boards at St. Michael's Hospital, Unity Health Toronto (REB#18‐196; approved 1 November 2018) and the Centre for Addiction and Mental Health (REB#156/2018; approved 19 December 2018) in Toronto, Canada. All participants provided either written or verbal informed consent to participate; access to an interpreter was available to facilitate understanding and a capacity‐to‐consent questionnaire was available for use by study staff as needed.

### 2.5 Data analysis

Grounded theory and inductive thematic analysis guided the interpretation of transcripts.^50^, ^51^, ^52^ This approach allowed for analysis of both themes informed by existing literature, such as behavioural economics and self‐determination theory, as well as themes that emerged independently, from the data itself. Coding was completed by three rigorously trained researchers, using an established methodology.^53^, ^54^, ^55^ Two separate codebooks and databases were developed for CATCH‐FI participants and for CATCH service providers and key informants, respectively. To develop the CATCH‐FI participant codebook, three researchers independently coded six transcripts and collectively reviewed results to identify a set of key codes. Once consensus was achieved, a codebook was developed and iteratively updated, guiding the coding of these remaining transcripts. Similarly, to develop the CATCH service provider and key informant codebook, two researchers independently coded the focus group transcript and three researchers independently coded two key informant interviews to identify a set of key codes from which to build the codebook. In instances where consensus on ultimate code application was not reached after an initial meeting between researchers, the PI was consulted and the consensus was achieved in a subsequent meeting. Inter‐rater reliability was assessed using Cohen's *κ* statistic,^56^ for which scores were substantial for both service user data (*κ* = 0.79) and service provider data (*κ* = 0.77); percent agreement on all codes was 99%. All interviews coded for the purpose of the codebook development were later recoded using the developed codebook. As coding progressed, codes were grouped into higher‐order themes. The PI and study staff met regularly to iteratively review and refine the coding framework and emerging themes. Data saturation was achieved in which no new codes or themes emerged from later interviews.

QSR International NVivo 9 qualitative analysis software was used to support data management, coding and analysis.

## 3 RESULTS

In investigating the perceived impact of FI on service engagement, health and well‐being, three primary themes emerged from participant narratives:

*Cash is king*: The first theme describes how FI directly facilitated engagement in a subgroup of CATCH‐FI participants.

*Not in it for the money*: The second theme describes how, for the majority of CATCH‐FI participants, FI was believed to be relatively less important for service engagement compared to other factors, such as an individual's intrinsic motivation and the quality of care offered.

*Money talks*: Finally, the third theme speaks to the universal agreement among study participants across stakeholder groups that FI directly support health and wellbeing by enabling access to basic needs and enjoyment of simple pleasures.

*‘Cash is king’: Financial incentives are a carrot and some are hungry*

Over one‐quarter of CATCH‐FI participants described that FI had or would have a direct impact on their decision to engage in care. A minority of participants FI enroled in the intervention arm described instances of engaging with their case managers with the primary intention of obtaining the incentive: ‘I thought I should make sure that I stay in touch… I knew that I would get an e‐transfer, so it worked’ (P4337). Often, these participants described FI as an effective externally motivating reinforcement of their overall efforts to engage in an ongoing manner: ‘the finances helped keep me going and doing it, so I guess it would be the consistency of [engaging] that was impacted’ (P4081). As one CATCH‐FI participant enroled in the usual care arm who was asked about the hypothetical influence of FI summarized, ‘it gives motivation… something to gain that would motivate people to show up more often than not’ (P4071). Those who decided to engage because of the FI went on to describe an improved overall care experience as a result:

I found that the incentive kept me in touch with someone that was helping me. It made me feel better because I knew someone was there. It's like, if the $20 thing wasn't there, I wouldn't have made that phone call, I know I would've felt more distanced from [case manager] and the program and like life itself… But I found the frequent contact kept my head more positive. (P4010)

The majority of CATCH service providers and key informants similarly believed that FI did or would likely directly influence service engagement (*n* = 7/12). This was primarily due to the perceived universal utility of a financial gain and specific lack of financial capital among the study population. As one individual described,

I think clients who are on the street… they have very few rewards and perks in their life, and so it makes sense to me to figure out what is it that they need and want. And one of the things that everybody needs and wants is…some level of finances… so it makes sense to me that we consider this as an option. (SP2)

*‘Not in it for the money’: The relative value of financial* incentives

The majority of CATCH‐FI participants indicated that FI was not or would not be directly associated with their decision to engage with the intervention (*n* = 16/22). These participants described feeling appreciative of the incentives, highlighting that an FI was useful but nonessential; they frequently referred to the incentives as a ‘bonus’ or ‘reward’. As one participant summarized, receiving an FI ‘didn't influence’ their decision to engage ‘because I would have done it regardless. But when I found out there was one, I was very happy’ (P4061).

CATCH‐FI participants described two relatively more meaningful facilitators of service engagement: (i) intrinsic motivation, at the service user level; and (ii) quality of care, at the service provider level.

*Intrinsic motivation matters more*: Many CATCH‐FI participants in the intervention arm highlighted the importance of intrinsic motivation in their decision to follow up with their case managers and remain engaged in their care plan. These participants explained that their motivations were internal and primarily focused on improving their immediate circumstances of homelessness and poor health—‘get[ing] out of the shelter system, find myself an apartment, try to get back to work, get off [financial assistance]’ (P4025)—in addition to the overarching desire for personal improvement. As one individual described, their decision to engage ‘wasn't for the money. It was for the better of me’ (P4106). Others echoed that FI was relatively less meaningful compared to this internal drive: [Financial incentives] are not like the pinnacle of why I'm doing it. I'm trying to better myself… to get out of this… I don't want to be stuck’ (P4068). For this subgroup of CATCH‐FI participants, intrinsic motivation was paramount in their decision to engage: the ‘desire to get better, to get out of where I am. That is core’ (P4092). CATCH‐FI participants enroled in the usual care arm echoed this perspective, with one individual suggesting ‘any person who wants help is probably going to seek the help whether there's a financial incentive or not’ (P4126).

*Quality of care is a stronger hook*: For other CATCH‐FI participants in the intervention arm, it was the quality of care provided by the programme as a whole and by individual staff members that primarily influenced their decision to engage in care. As one individual described the programme, ‘this is a lifesaver… my life was saved… that's why I would have done it regardless’ (P4061). This subgroup suggested they were particularly engaged by CATCH service providers who actively listened and addressed their needs. One participant explained how they ‘already enjoyed meeting with [case manager] and having someone I could talk to… who actually understood and didn't try to cut me off… someone who actually looked at me’ (P4004); and another expanded, ‘mainly I went to see him because he provided results and helped me with my healthcare… because of him, I would have gone to see him anyway. Every week, regardless’ (P4023). CATCH‐FI participants enroled in the usual care arm similarly echoed their counterparts' perspective in articulating that an FI ‘would be nice, but that wouldn't have been more of an incentive – I mean, at least not for me because I was getting so much out of the program’ (P4057).

In contrast to the majority of CATCH‐FI participants speaking to relatively more important facilitators of engagement, only one frontline service provider or key informant similarly noted that FI might be a less impactful motivator:

The financial incentive I think is very important for certain kinds of clients who that is their focus, but I think a lot of people are just really hungry for that engagement in someone that's looking out for them and they trust. (FG3)

This group of stakeholders did, however, articulate a scenario in which FI could be used to support initial engagement while service providers work toward building trust through the sustained high quality of care. For example, CATCH service providers and key informants described how the prospect of an FI can support initial engagement by ‘tip[ping] the balance’ (SP5) and ‘provid[ing] a moment in time to help with building that connection’ (SP1) to ‘get them through the door for the first part. Then the second part would be following through with what they've identified they want help with’ (SP3).

*‘Money talks’: The utility of financial* incentives *in supporting health and well‐being*

Participants across stakeholder groups agreed that FI directly supports health and well‐being; all 22 CATCH‐FI participants described examples of how receiving FI did or would have improved their health status with five of 12 CATCH‐FI service providers and key informants providing similar perspectives. Overall, study participants described two primary mechanisms by which FI was used to support their health and well‐being: the fulfilment of *basic needs* and *simple pleasures*.

*Addressing basic needs*: Study participants, including over two‐thirds of CATCH‐FI participants, described how FI afford more opportunities to meet basic needs that directly support health and well‐being, such as satisfying hunger, taking prescribed medications and reducing stress. As one CATCH‐FI participant in the intervention arm described, ‘It had a mental health [impact], yes. And yeah physical health “cause I was able to eat… I was going like entire days without food”’ (P4010). Other CATCH‐FI participants enroled in the intervention arm described using the FI they received for food, medications, supplies, public transportation fare and to pay bills: basic needs that ‘reduces stress because I'm able to get the little things I need… that money allowed me to pay my cell phone bill and it left me a small amount to get the groceries I needed for the week’ (P4249). The experience was impactful for CATCH‐FI participants, suggesting that the intervention ‘really helps financially when you're on a very low fixed income… I use that money to be able to help me survive’ (P4061). CATCH‐FI participants assigned to the usual care arm similarly described that receiving FI would be useful in directly enabling individuals to meet basic needs that are essential to health and well‐being:

I do think [financial incentives] are really, really… useful because there comes a point when you're too broke to do anything. Like it's all well and good that you've made all these appointments and you have all these dreams but when it really comes down to the nitty gritty, if you don't have money, you can't afford to do it. You can't afford to go see your doctor because you can't afford bus fare. You can't afford to get a job because you can't afford interview clothes. (P4031)

CATCH service providers and key informants similarly highlighted the perceived utility of FI in meeting basic needs to support health and well‐being: ‘I think that realistically, incentives do support people being able to maintain their wellness and engage in care’ (HP5). For example, CATCH service providers described how incentives in this study were used practically by CATCH‐FI participants ‘to get from one appointment to the next and to accomplish some of these very important tasks that they need to do: to get their ID, or meet up with a doctor… get their medication’ (FG5). Overall, this stakeholder group generally agreed that the use of FI can positively support the health and well‐being of this population insofar as ‘anything that may be able to help people stabilize those basic needs is critical to supporting their ability to engage in slightly less pressing health and social care’ (HP6).

*Allowing for simple pleasures*: Similar to meeting basic needs, FI allowed CATCH‐FI participants to experience simple pleasures, small gains or rewards that directly improved their mental health and well‐being by relieving stress or giving them an enjoyable experience to anticipate. CATCH‐FI participants enroled in the intervention arm described the positive emotions experienced after spending their incentives on small items, such as coffee, restaurant food and cigarettes; one individual reported purchasing illegal drugs with the incentive. Overall, participants in this study consistently described perceived positive impacts on mental health and well‐being as a direct result of receiving an FI. For example, one individual described their experience as follows:

It makes you feel better when you have money to spend on yourself… With the financial incentive, I found it gave me something to look forward to. It's like, ‘Yeah, I can have McDonald's and give myself a little treat’… It helped better my life. It helped pull me ahead, basically. (P4010)

CATCH‐FI participants in the usual care arm similarly perceived that a financial incentive would improve their mental health and well‐being, suggesting that ‘if you don't feel so destitute and broke all the time, it's gonna lift up your spirits, for sure’ (P4057). While many CATCH‐FI participants spoke of the importance of simple pleasures and their impact on well‐being, CATCH service providers and key informants did not describe this potential utility of FI for the study population.

## 4 DISCUSSION

This qualitative study explored stakeholder perspectives on the use of FIs to support service engagement and improve health and well‐being among adults experiencing homelessness and mental illness in a large urban centre in Canada. Consistent with prior research, our findings suggest that FI may successfully facilitate service engagement for some service users. A subset of service users in this study described the prospect of immediate financial gain as an externally motivating factor that encouraged continued contact with service providers. These findings are consistent with behavioural health economics principles, which suggest extrinsically motivated individuals and those particularly biased toward present and immediate rewards may be especially likely to respond to FI.^17^, ^27^, ^28^

Our findings are unique, however, in identifying that the majority of service users perceived FI to be less impactful than other key facilitators of service engagement. In particular, service users highlighted the relatively greater value of intrinsic motivation, which renders FI extraneous. This finding aligns with self‐determination theory^29^, ^30^ and previous literature, suggesting intrinsic motivation is a key ingredient in initiating and especially sustaining health behaviours.^31^, ^32^, ^33^, ^57^, ^58^

Our study findings also speak to the importance of quality of care, which emerged as another key facilitator of service engagement across all stakeholder groups. Many service users in this study described how perceived quality of programming and quality of relationships with case managers independently motivated their decision to engage above and beyond the influence of FI. This finding reiterates the central role of high‐quality, client‐centred and relationship‐based models of care in sustaining engagement in this population. And while our findings indicate quality in and of itself motivates engagement, literature also suggests high‐quality services can support engagement by facilitating intrinsic motivation.^31^, ^57^, ^59^ Within the context of incentive‐based interventions, further research elucidating the relationship between quality of care and motivation in service engagement is warranted.

Taken together, particularly among the subset of service users who might want or need an extrinsic motivator to support initial engagement, our findings suggest that FI may offer an effective opportunity to initially engage or ‘hook’ some service users; and that a complementary focus on the quality of care by service providers may further help sustain engagement. The need to enhance sustainability is consistent with findings from a recent qualitative study in which FI were associated with initial motivation to engage in low‐barrier human immunodeficiency virus care but were less effective in facilitating sustained engagement^60^; and reviews of existing quantitative evidence indicate FI is particularly effective for singular behaviours^61^ and short‐term engagement and behaviour change.^18^

Beyond the direct influence on service engagement, participant narratives consistently described clear, positive impacts of FI on health and well‐being. Primarily, participants described that FI enabled them to afford basic needs (e.g., food, transportation fare, bill payments) and to enjoy simple pleasures (e.g., coffee, entertainment) that were in turn perceived to directly support their physical and mental health and overall sense of well‐being. That only one service user reported using their FI to purchase illegal drugs is consistent with previous research^62^, ^63^ and noteworthy, considering that a key criticism of this engagement strategy is the potential to exacerbate substance use.^18^, ^36^ As suggested by service providers in this study, it is possible that helping service users fulfil basic survival and immediate needs may additionally support service engagement. Given the limited literature on this topic, further research into the mechanisms by which FI may directly and indirectly support engagement is needed.

A final notable finding in this study is the divergence in stakeholder perspectives. Overall, service providers consistently expressed the view that FI would likely support service engagement of adults experiencing homelessness and mental health needs. This perspective reflected the assumption that the perceived utility of FI, specifically among this population who lack basic health, social and financial capital, was or would be high and sufficiently motivating to independently drive service engagement. This stakeholder perspective stands in significant contrast to the majority perspective among service user participants, two‐thirds of whom articulated that FI alone were not or would not be a significant influence on their decision to engage in care. This divergence suggests stakeholders may differentially perceive utility and ascribe value to FI. Further research and a better understanding of service user perspectives on the extent to which, how and why FI are useful will be essential to designing and implementing acceptable client‐centred and context‐specific interventions to promote engagement.

## 5 STRENGTHS AND LIMITATIONS

This study is part of a larger mixed‐methods RCT and our findings are strengthened by a methodologically rigorous design. Our resulting rich, multistakeholder narrative data add high‐quality evidence to an underdeveloped literature base and significantly improve our understanding of the perceived impact of using FI to support engagement, health and well‐being in an underserved population. Our findings from a large urban centre are context‐specific and reflect the experiences and potential biases of study participants. Nonetheless, our findings may be helpful to other programmes or jurisdictions considering alternative strategies to improve engagement among people experiencing homelessness and mental illness and other underserved populations.

Study limitations include the cross‐sectional design and the narrow demographics of the qualitative sample, which was primarily male, Caucasian and at least high school‐educated. Differences between this qualitative sample and the overall trial sample may reflect, in part, the purposive sampling strategy. The study team made several attempts to ensure a representative sample, including through the expansion of eligibility criteria, but recruitment remained challenged by reasons such as poor health status, nonresponse to invitations to participate and refusal to participate. Although sample characteristics were generally representative of the larger trial population, participants with different sociodemographic, health status and service engagement profiles may perceive and experience FI differently.

### 5.1 Future research

Given the paucity of research in this area, and the need for interventions to improve service engagement of underserved and disadvantaged populations, further research is warranted to compare and contrast various engagement strategies using rigorous methods and large study samples. In particular, additional research should investigate how FI may be timed and dosed to enhance other facilitators of service engagement, such as intrinsic motivation and quality of care. Further, in addition to high‐quality evidence on effectiveness, more research is needed on the ethicality, acceptability and feasibility of this approach within and across diverse subpopulations and service settings. Ethical concerns, including perceived coercion and unintended consequences, remain key barriers that have resulted in underrepresentation in research and practice of a promising intervention.

### 5.2 Conclusions

Key stakeholders described that FIs may improve service engagement among some adults experiencing homelessness and mental illness. Stakeholders also described that FIs positively impact health and well‐being, easing financial stress and enabling deeper attention to individual health needs. Findings from this study add to an underdeveloped literature base on stakeholder perspectives and experiences of using FIs to improve engagement, health and well‐being of homeless adults with mental health needs.

## AUTHOR CONTRIBUTIONS

All authors contributed significantly to this manuscript. Nadine Reid led the interpretation of results and drafted the manuscript. Rebecca Brown and Cheryl Pedersen participated in study coordination, data collection and analysis and editing of the manuscript. Nicole Kozloff and Alexandra Sosnowski contributed meaningful edits to the manuscript. Vicky Stergiopoulos is the primary investigator who overall conceived of and supervised this study and drafting of the manuscript.

## Data Availability

Data are available through the corresponding author upon request in accordance with Unity Health Institutional policies.

## References

[1] Fazel S , Geddes JR , Kushel M . The health of homeless people in high‐income countries: descriptive epidemiology, health consequences, and clinical and policy recommendations. Lancet. 2014;384(9953):1529‐1540. 10.1016/S0140-6736(14)61132-6 25390578 PMC4520328

[2] Hwang SW . Homelessness and health. CMAJ. 2001;164(2):229‐233. 11332321 PMC80688

[3] Stergiopoulos V , Cusi A , Bekele T , et al. Neurocognitive impairment in a large sample of homeless adults with mental illness. Acta Psychiatr Scand. 2015;131(4):256‐268. 10.1111/acps.12391 25604122

[4] Batchelor P , Kingsland J . Improving the health of the homeless and how to achieve it within the new NHS architecture. Int J Environ Res Public Health. 2020;17(11):4100. 10.3390/ijerph17114100 PMC7312815 32521822

[5] Cernadas A , Fernandez A . Healthcare inequities and barriers to access for homeless individuals: a qualitative study in Barcelona (Spain). Int J Equity Health. 2021;20:84. 10.1186/s12939-021-01409-2 33743729 PMC7980324

[6] Dixon LB , Holoshitz Y , Nossel I . Treatment engagement of individuals experiencing mental illness: review and update. World Psychiatry. 2016;15(1):13‐20. 10.1002/wps.20306 26833597 PMC4780300

[7] Stafford A , Wood L . Tackling health disparities for people who are homeless? Start with social determinants. Int J Environ Res Public Health. 2017;14(12):1535. 10.3390/ijerph14121535 PMC5750953 29292758

[8] Liu M , Hwang SW . Health care for homeless people. Nat Rev Dis Primers. 2011;7(5), 10.1038/s41572-020-00241-2 33446661

[9] Haddad PM , Brain C , Scott J . Nonadherence with antipsychotic medication in schizophrenia: challenges and management strategies. Patient Relat Outcome Meas. 2014;5:43. 10.2147/PROM.S42735 25061342 PMC4085309

[10] Kreyenbuhl J , Nossel IR , Dixon LB . Disengagement from mental health treatment among individuals with schizophrenia and strategies for facilitating connections to care: a review of the literature. Schizophr Bull. 2009;35(4):696‐703. 10.1093/schbul/sbp046 19491314 PMC2696379

[11] George M , Manuel JI , Gandy‐Guedes ME , McCray S , Negatu D . “Sometimes what they think is helpful is not really helpful”: understanding engagement in the Program of Assertive Community Treatment (PACT). Community Ment Health J. 2016;52:882‐890. 10.1007/s10597-015-9934-9 26335712

[12] Shaw J , Conover S , Herman D , et al. Critical time intervention for severely mentally ill prisoners (CRISP): a randomized trial. Health Service Deliv Res. 2017;5(8):1‐138. 10.3310/hsdr05080 28252895

[13] Bassett IB , Wilson D , Taaffe J , Freedberg KA . Financial incentives to improve progression through the HIV treatment cascade. Curr Opin HIV AIDS. 2015;10(6):451‐463. 10.1097/COH.0000000000000196 26371461 PMC4699403

[14] Lagarde M , Haines A , Palmer N . Conditional cash transfers for improving uptake of health interventions in low‐ and middle‐income countries: a systematic review. JAMA. 2007;298(16):1900‐1910. 10.1001/jama.298.16.1900 17954541

[15] Operario D , Kuo C , Sosa‐Rubi SG , Galarraga O . Conditional economic incentives for reducing HIV risk behaviors: integration of psychology and behavioural economics. Health Psychol. 2013;32(9):932‐940. 10.1037/a0032760 24001243 PMC4118666

[16] Volpp KG , John LK , Troxel AB , Norton L , Fassbender J , Lowenstein G . Financial incentive‐based approaches for weight loss: a randomized trial. JAMA. 2008;300(2):2631‐2637. 10.1001/jama.2008.804 19066383 PMC3583583

[17] Carminati L . Behavioural economics and human decision making: Instances from the health care system. Health Policy. 2020;124(6):659‐664. 10.1016/j.healthpol.2020.03.012 32386789

[18] Giles EL , Robalino S , McColl E , Sniehotta FF , Adams J . The effectiveness of financial incentives for health behaviour change: systematic review and meta‐analysis. PLoS One. 2014;9(3):e90347. 10.1371/journal.pone.009347 24618584 PMC3949711

[19] Wu W . The relationship between incentives to learn and Maslow's Hierarchy of Needs. Physics Procedia. 2012;24(Part B):1335‐1342. 10.1016/j.phpro.2012.02.199

[20] Post E , Cruz M , Harman J . Incentive payments for attendance at appointments for depression among low‐income African Americans. Psychiatr Serv. 2006;57(3):414‐416. 10.1176/appi.ps.57.3.414 16525004

[21] Stanley IH , Chu C , Brown TA , Sawyer KA , Joiner TE Jr . Improved clinical functioning for patients receiving fee discounts that reward treatment engagement. J Clin Psychol. 2016;72(1):15‐21. 10.1002/jclp.22236 26613565

[22] Priebe S , Yeeles K , Bremner S , et al. Effectiveness of financial incentives to improve adherence to maintenance treatment with antipsychotics: cluster randomised controlled trial. BMJ. 2013;347:f5847. 10.1136/bmj.f5847 24100934 PMC3805491

[23] Messina N , Farabee D , Rawson R . Treatment responsivity of cocaine‐dependent patients with antisocial personality disorder to cognitive‐behavioral and contingency management interventions. J Consult Clin Psychol. 2003;71(2):320‐329. 10.1037/0022-006x.71.2.320 12699026

[24] Davis DR , Kurti AN , Skelly JM , Redner R , White TJ , Higgins ST . A review of the literature on contingency management in the treatment of substance use disorders, 2009‐2014. Prev Med. 2016;92:36‐46. 10.1016/j.ypmed.2016.08.008 27514250 PMC5385000

[25] Baggett TP , Chang Y , Yaqubi A , McGlave C , Higgins ST , Rigotti NA . Financial incentives for smoking abstinence in homeless smokers: a pilot randomized controlled trial. Nicotine Tob Res. 2018;20(12):1442‐1450. 10.1093/ntr/ntx178 29059442 PMC6236071

[26] Rash CJ , Petry NM , Alessi SM . A randomized trial of contingency management for smoking cessation in the homeless. Psychol Addict Behav. 2018;32(2):141‐148. 10.1037/adb0000350 29461070 PMC5858980

[27] Mogler BK , Shu SB , Fox CR , et al. Using insights from behavioral economics and social psychology to help patients manage chronic diseases. J Gen Intern Med. 2013;28(6):711‐718. 10.1007/s11606-012-2261-8 23229906 PMC3631076

[28] Rice T . The behavioural economics of health and health care. Annu Rev Public Health. 2013;34:431‐447. 10.1146/annurev-publhealth-031912-114353 23297657

[29] Ryan RM , Deci EL . Self‐determination theory and the facilitation of intrinsic motivation, social development, and well‐being. Am J Psychol. 2000;55:68‐78. 10.1037//0003-066x.55.1.68 11392867

[30] Vallerand RJ . Deci and Ryan's self‐determination theory: a view from the hierarchical model of intrinsic and extrinsic motivation. Psychol Inquiry. 2000;11(4):312‐318.

[31] Patrick H , Williams GC . Self‐determination theory: its application to health behavior and complementarity with motivational interviewing. Int J Behav Nutr Phys Act. 2012;9:18. 22385676 10.1186/1479-5868-9-18 PMC3323356

[32] Chan GHY , Lo TW , Tam CHL , Lee GKW . Intrinsic motivation and psychological connectedness to drug abuse and rehabilitation: the perspective of self‐determination. Int J Environ Res Public Health. 2019;16:1934. 10.3390/ijerph16111934 PMC6603877 31159227

[33] DiClemente CC . Motivation for change: implications for substance abuse treatment. Psychol Sci. 1999;10(3):209‐213. 10.1111/1467-9280.00137

[34] Giles EL , Robalino S , Sniehotta F , Adams J , Mccoll E . Acceptability of financial incentives for encouraging uptake of healthy behaviours: a critical review using systematic methods. Prev Med. 2015a;73:1. 10.1016/j.ypmed.2014.12.029 25600881

[35] Giles EL , Sniehotta FF , McColl E , Adams J . Acceptability of financial incentives and penalties for encouraging uptake of health behaviours: focus groups. BMC Public Health. 2015b;5:58. 10.1186/s12889-015-1409-y PMC4318173 25636330

[36] Hoskins K , Ulrich CM , Chinnick J , Buttenheim AM . Acceptability of financial incentives for health‐related behavior change: an updated systematic review. Prev Med. 2019;126:105762. 10.1016/j.ypmed.2019.105762 31271816

[37] Noordraven EL , Schermer MHN , Blanken P , Mulder CL , Wierdsma AI . Ethical acceptability of offering financial incentives for taking antipsychotic depot medication: patients' and clinicians' perspectives after a 12‐month randomized controlled trial. BMC Psychiatry. 2017;17(1):313‐468. 10.1186/s12888-017-1485-x 28851345 PMC5576283

[38] Priebe S . Acceptability of offering financial incentives to achieve medication adherence in patients with severe mental illness: a focus group study. J Med Ethics. 2010;36(8):463‐468. 10.1136/jme.2009.035071 20581423 PMC2976611

[39] Lamanna D , Stergiopoulos V , Durbin J , O'campo P , Poremski D , Tepper J . The impact of financial incentives on service engagement among adults experiencing homelessness and mental illness: a pragmatic trial protocol. Front Psychiatry. 2021;12:1316‐1364. 10.3389/fpsyt.2021.722485 PMC8369574 34413804

[40] Lamanna D , Stergiopoulos V , Durbin J , O'Campo P , Poremski D , Tepper J . Promoting continuity of care for homeless adults with unmet health needs: the role of brief interventions. Health Soc Care Community. 2018;26(1):56‐64. 10.1111/hsc.12461 28569397

[41] Stergiopoulos V , Gozdzik A , Cohen A , et al. Evaluating the impact of a critical time intervention adaptation on health care utilization among homeless adults with mental health needs in a large urban centre. Can J Psychiatry. 2021;12:e0182157. 10.1177/0706743721996114 PMC8811242 33611924

[42] Stergiopoulos V , Gozdzik A , Nisenbaum R , et al. The effect of brief case management on emergency department use of frequent users in mental health: findings of a randomized controlled trial. PLoS One. 2018;12(8):774‐784 e0182157. 10.1371/journal.pone.0182157 PMC5542632 28771524

[43] Stergiopoulos V , Gozdzik A , Nisenbaum R , et al. Bridging hospital and community care for homeless adults with mental health needs: outcomes of a brief interdisciplinary intervention. Can J Psychiatry. 2018;63(11):774‐784. 10.1177/0706743718772539 29716396 PMC6299183

[44] Stergiopoulos V , Gozdzik A , Tan de Bibiana J , et al. Integrating hospital and community care for homeless people with unmet mental health needs: program rationale, study protocol and sample description of a brief multidisciplinary case management intervention. Int J Ment Health Addiction. 2017;15(2):362‐378. 10.1007/s11469-017-9731-5

[45] Herman D , Conover S , Felix A , Nakagawa A , Mills D . Brief case management versus usual care for frequent users of emergency departments: the Coordinated Access to Care from Hospital Emergency Departments (CATCH‐ED) randomized controlled trial. BMC Health Serv Res. 2016;16(1):432‐312. 10.1186/s12913-016-16666-1 27557705 PMC4997752

[46] Tomita A , Herman DB . Critical time intervention: an empirically supported model for preventing homelessness in high risk groups. J Primary Prevent. 2007;28:295‐312. 10.1007/s10935-007-0099-3 17541827

[47] Tomita A , Herman DB . The impact of critical time intervention in reducing psychiatric rehospitalization after hospital discharge. Psychiatr Serv. 2012;63(9):935‐937. 10.1176/appi.ps.201100468 22810163 PMC3989527

[48] Khan B , Reid N , Brown R , Kozloff N , Stergiopoulos V . Engaging adults experiencing homelessness in recovery education: A qualitative analysis of individual and program level enabling factors. Front Psychiatry. 2020;11:779. 10.3389/fpsyt.2020.00779 32848944 PMC7424067

[49] Reid N , Khan B , Soklaridis S , Kozloff N , Brown R , Stergiopoulos V . Mechanisms of change and participant outcomes in a Recovery Education Centre for individuals transitioning from homelessness: a qualitative evaluation. BMC Public Health. 2020;20:497. 10.1186/s12889-020-08614-8 32295561 PMC7161283

[50] Glaser BG , Strauss A . Discovery of Grounded Theory: Strategies for Qualitative Research. Sociology Press; 1967.

[51] Birks M , Mills J . Grounded Theory: A Practical Guide. SAGE Publications; 2015.

[52] Braun V , Clark V . Using thematic analysis in psychology. Qual Res Psychol. 2006;3(2):77‐101. 10.1191/1478088706qp063oa

[53] Devotta K & Pedersen C Coding qualitative data: working with a team of coders. CRICH Survey Research Unit Methodology Bits; 2015. November 30, 2021. https://hdl.handle.net/1807/70247

[54] Patton M . Qualitative Research & Evaluation Methods. 4th ed. Sage Publications; 2015.

[55] Saldana J . The Coding Manual for Qualitative Researchers. 2nd ed. Sage Publications; 2015.

[56] McHugh ML . Interrater reliability: the kappa statistic. Biochem Med. 2012;22(3):276‐282. PMC3900052 23092060

[57] Zeldman A , Ryan RM , Fiscella K . The importance of self‐mastery in enhancing quality of life and social participation of individuals experiencing homelessness: results of a mixed‐method study. Soc Indic Res. 2020;148:491‐515. 10.1007/s11205-019-02211-y

[58] Zeldman A , Ryan R , Fiscella K . Motivation, autonomy support, and entity beliefs: their role in methadone maintenance treatment. J Soc Clin Psychol. 2004;23:675‐696.

[59] Klag SML , Creed P , O'Callaghan F . Early motivation, well‐being, and treatment engagement of chronic substance users undergoing treatment in a therapeutic community setting. Subst Use Misuse. 2010;45:1112‐1130. 20441454 10.3109/10826080903499562

[60] Beima‐Sofie K , Begnel ER , Golden MR , Moore A , Ramchandani M , Dombrowski JC . “It's me as a person, not me the disease” Patient perceptions of an HIV care model designed to engage persons with complex needs. AIDS Patient Care STDS. 2020;34(6):267‐273. 10.1089/apc.2019.0310 32484744 PMC7262637

[61] Merrick EL , Hodgkin D , Horgan CM . Incentives to shape health behaviors: how can we make them more person‐centred? J Workplace Behav Health. 2014;29(1):19‐31. 10.1080/15555240.2014.868721

[62] Festinger DS , Dugosh KL , Kirby KC , Seymour BL . Contingency management for cocaine treatment: cash vs. vouchers. J Subst Abuse Treat. 2014;47(2):168‐174. 10.1016/j.sat.2014.03.001 24746956 PMC4504189

[63] Festinger DS , Marlowe DB , Croft JR , et al. Do research payments precipitate drug use or coerce participation? Drug Alcohol Depend. 2005;78(3):275‐281. 15893158 10.1016/j.drugalcdep.2004.11.011

